# A High-Precision Deep Learning Algorithm to Localize Idiopathic Ventricular Arrhythmias

**DOI:** 10.3390/jpm12050764

**Published:** 2022-05-09

**Authors:** Ting-Yung Chang, Ke-Wei Chen, Chih-Min Liu, Shih-Lin Chang, Yenn-Jiang Lin, Li-Wei Lo, Yu-Feng Hu, Fa-Po Chung, Chin-Yu Lin, Ling Kuo, Shih-Ann Chen

**Affiliations:** 1 Heart Rhythm Center, Division of Cardiology, Department of Medicine, Taipei Veterans General Hospital, Taipei 11217, Taiwan; tingyungchang@gmail.com (T.-Y.C.); sasuke9301108@hotmail.com (C.-M.L.); linyennjiang@gmail.com (Y.-J.L.); gyrus1975@gmail.com (L.-W.L.); huhuhu0609@gmail.com (Y.-F.H.); marxtaiji@gmail.com (F.-P.C.); clouaa@gmail.com (C.-Y.L.); kl19860209@gmail.com (L.K.); epsachen@ms41.hinet.net (S.-A.C.); 2 Institute of Cardiovascular Research, National Yang Ming Chiao Tung University, Taipei 11221, Taiwan; 3 Institute of Clinical Medicine, National Yang Ming Chiao Tung University, Taipei 11221, Taiwan; 4 Department of Nursing, National Taipei University of Nursing and Health Sciences, Taipei 112303, Taiwan; 5 Department of BioMedical Engineering, National Cheng Kung University, Tainan City 701401, Taiwan; gosienna@gmail.com; 6 Cardiovascular Center, Taichung Veterans General Hospital, Taichung 40705, Taiwan

**Keywords:** machine learning, ventricular arrhythmia, localization, catheter ablation

## Abstract

Background: An accurate prediction of ventricular arrhythmia (VA) origins can optimize the strategy of ablation, and facilitate the procedure. Objective: This study aimed to develop a machine learning model from surface ECG to predict VA origins. Methods: We obtained 3628 waves of ventricular premature complex (VPC) from 731 patients. We chose to include all signal information from 12 ECG leads for model input. A model is composed of two groups of convolutional neural network (CNN) layers. We chose around 13% of all the data for model testing and 10% for validation. Results: In the first step, we trained a model for binary classification of VA source from the left or right side of the chamber with an area under the curve (AUC) of 0.963. With a threshold of 0.739, the sensitivity and specification are 90.7% and 92.3% for identifying left side VA. Then, we obtained the second model for predicting VA from the LV summit with AUC is 0.998. With a threshold of 0.739, the sensitivity and specificity are 100% and 98% for the LV summit. Conclusions: Our machine learning algorithm of surface ECG facilitates the localization of VPC, especially for the LV summit, which might optimize the ablation strategy.

## 1. Introduction

Patients with a high burden of ventricular arrhythmia (VA) such as ventricular tachycardia (VT) or ventricular premature complex (VPC) are more prone to experience deterioration of left ventricular ejection fraction (LVEF) and incidence of heart failure (HF) as well as sudden cardiac death (SCD) [1]. Catheter ablation (CA) has emerged as a therapeutic method to treat VA, with a low procedural complication risk and a high success rate of cure [2]. Although most idiopathic VA originates from the right ventricular outflow tract (RVOT) or left coronary cusps, in some cases, the VA might originate from the LV summit. VA originating from this region could be more challenging for catheter ablation, and the failure rate in previous reports was relatively high due to the coverage of epicardial fat and the proximity of main coronary arteries if the percutaneous epicardial approach is planned [3]. VA arising from these locations mostly presents with a unique pattern on 12-lead surface ECG [4,5]. Distinguishing the right or left side of origin before the procedure is helpful in terms of guiding access for catheter ablation. Furthermore, an accurate prediction of VA origins can optimize the strategy of ablation, such as the LV summit, reduce procedural time, and avoid pre-procedural complications. Previous studies proposed several methods to estimate the origins of ventricular arrhythmias [6,7,8,9,10,11,12]. However, the accuracy has been limited by the number of patient studies, generalizability of the models, and efficiency of application [13]. On the other hand, trained by finding subclinical patterns in huge datasets, Artificial Intelligence (AI) has transformed the ECG into a screening tool and predictor of cardiac and non-cardiac diseases [14]. Furthermore, Hussain et al. demonstrated that AI could also be integrated into a cyber-physical cardiac monitoring system for stroke management and assist in the quantitative evaluation of neurological outcomes after stroke [15,16]. In this study, we aimed to develop a deep learning model to predict VA with origins on both sides of the ventricle and distinguish the origin of LV summits in particular, with clinical-grade precision.

The contribution of the current study was as followed:

(1) This present study has the largest cohort for deep learning to localize idiopathic VAs.
(2) Among the studies trying to solve VAs localization with deep learning or machine learning methods, our study has the largest number of cases for testing.
(3) We are among the first studies to used 2D-CNN for VAs localization.
(4) With violin plot, we can identify that VAs from the cusp area are the main source of errors.
(5) This is the first study for a deep learning algorithm to differentiate VAs from LV summit, with a sensitivity of 100% and a specificity of 98%.
(6) Accurate prediction of VAs from LV summit before the procedure would be helpful for the operator to optimize the whole procedure.

## 2. Method

### 2.1. Study Design

Between January 2015 and December 2020, a total of 397 patients with symptomatic and drug-refractory idiopathic VAs referred to Taipei Veterans General Hospital for ablation were enrolled. This study was approved by the institutional review board of the Taipei Veterans General Hospital (IRB:2022-03-001BC). The study was conducted by the Declaration of Helsinki. Patients with a medical history of cardiomyopathy and structural or congenital abnormalities were excluded. All of the 12-lead ECGs during the catheter ablation procedure had been recorded and stored on the LABSYSTEM™ Pro EP Recording System (Boston Scientific, Marlborough, MA, USA). The signals were recorded at a sampling frequency of 2000 Hz and were filtered with a low-frequency digital filter cutoff of 0.05 Hz and a high-frequency digital filter cutoff of 100 Hz. We manually identified the single VPC and exported it for further analysis. The VPC was initially identified as LVOT but shown to be outside of the cusp area after ablation was removed for simplicity.

**Figure 1:**
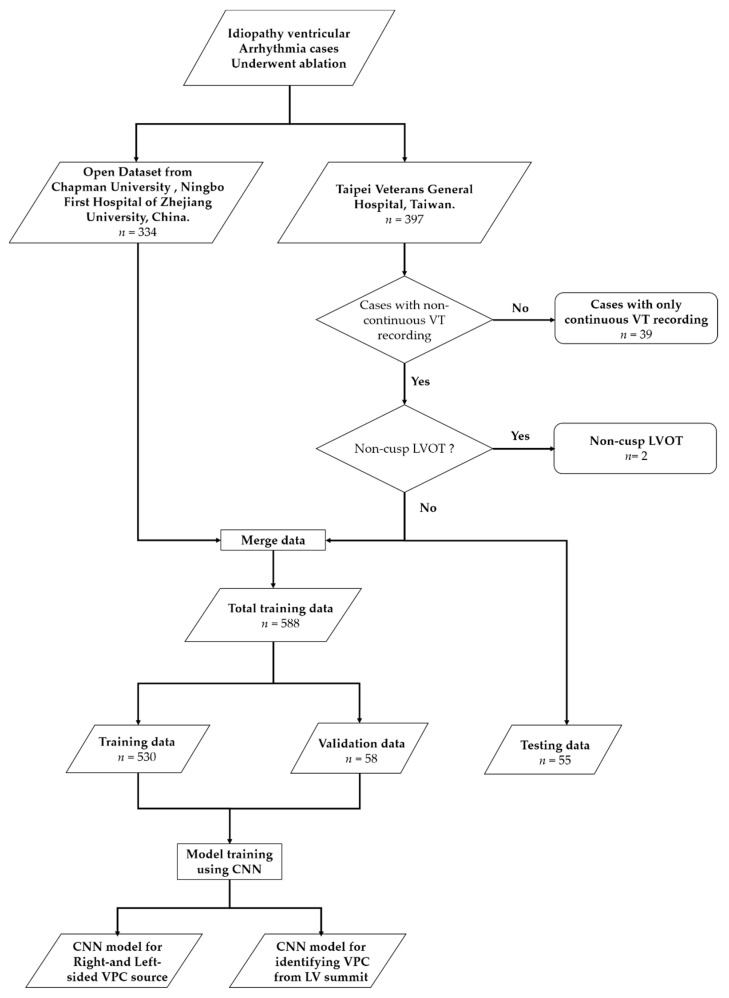
Overview of study design and data allocation.

Furthermore, to increase our training data size, we also included the online datasets presented in a previously published study [17]. This data set is composed of 334 patients which are gathered at Chapman University and Ningbo First Hospital of Zhejiang University, Ningbo, China. The main difference between this data set from ours is that it lacks VPCs other than ventricle outflow. The overview of the study design is shown in Figure 1.

### 2.2. Mapping and Ablation Procedure

**Figure 2:**
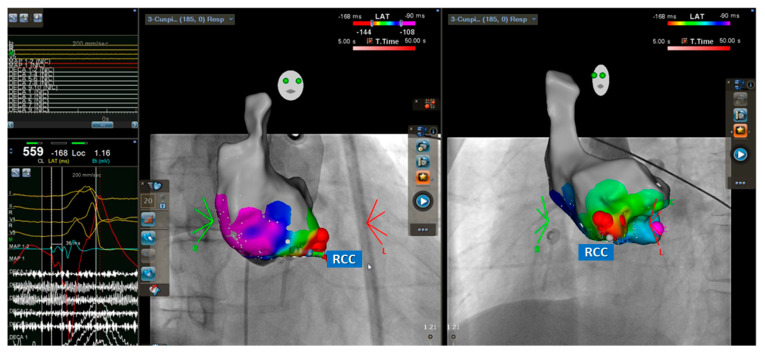
Activation map and fluoroscopic map for ventricular premature complex originating from the right coronary cusp (RCC). The local activation map showed the earliest activation site (−36 ms) at the RCC and catheter ablation in this area (red spots) could eliminate the ventricular arrhythmia.

The electrophysiological study, mapping, and RFCA were performed as described previously [3,18,19,20]. Antiarrhythmic agents (except amiodarone) were discontinued for a minimum of five half-lives before RFCA. We performed a standardized electrophysiological study for all patients in the fasting state without sedation. In the absence of spontaneous VA, rapid ventricular pacing, and/or programmed stimulation of up to three extrastimuli were attempted from the right ventricular apex. If VA was still not inducible, intravenous isoproterenol 1–5 μg/min was infused to achieve at least a 20% heart rate increment. If clinical VAs were not induced during pharmacological provocation, the induction protocol was repeated. The QRS morphologies of spontaneous and/or induced VAs were compared with those of clinical VAs. The localization of arrhythmogenic foci was detected conventionally or using a 3D mapping system (Ensite NavX, St. Jude Medical, Inc., St. Paul, MN, USA, or CARTO 3, Biosense Webster, Diamond Bar, CA, USA). To identify the origin and optimal ablation site of VA, we performed activation mapping, defined by the earliest local electrical signals, and/or pace mapping, aiming for at least 11 of 12 leads matching with clinical VAs. (Figure 2) During mapping of the left side, intravenous heparin was administered to maintain an activated clotting time of >250 s. Radiofrequency energy was delivered in a temperature-controlled mode at 50–60 °C with a pulse duration of 60 s for each point; maximal power was 50 W for the non-irrigated catheter and 30–35 W for the irrigated catheter with a maximum electrode-tissue interface temperature of 43 °C, targeting for an impedance decrease of 10 Ω. If the VA was suppressed within 30 s, radiofrequency energy would be maintained for a total of 120–300 s. Repeat mapping was performed if VA suppression and/or elimination were not observed. Acute procedural success was defined as the complete elimination of spontaneous or inducible VAs under the infusion of isoproterenol (up to 5 μg/min), following the same induction protocol for 30 min to exclude acute recurrences. The successful ablation site was defined by the point w an elimination of targeted VA with radiofrequency energy application.

The VA origin was defined as the ablation site where the VA/VPC was eliminated or suppressed by at least 80% of a burden if not eliminated. The classification of VA origins are shown in Table 1 [21]. VA origins were also classified into two groups: the right or left side of the heart. This was used for binary classification in machine learning. If several chambers were ablated, the elimination site was considered to be the origin except for the LV summit. The VA originated from the LV summit were according to the following criteria: (1) The earliest activation site within the LV summit (great cardiac vein/anterior interventricular vein (GCV/AIV) or epicardium) was identified based on fluoroscopy and electroanatomic map when VPCs were mappable. (2) For unmappable VA (e.g., VPCs that occurred too infrequently to allow for detailed activation mapping or were hemodynamically unstable), the best pace mapping sites, defined as 95% pace mapping score or 12/12 lead matching, were located within the LV summit region (GCV/AIV or epicardium) by assessing the response to ablation at the best pace mapping site with evidence of VA elimination [3].

### 2.3. Data Preprocessing

**Figure 3:**
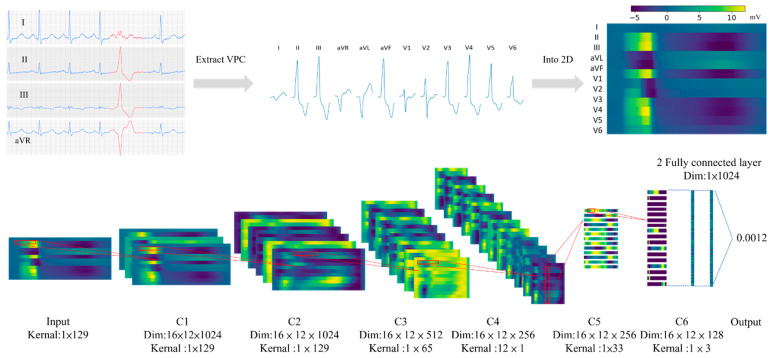
Data preprocessing and model structure demonstrated with feature maps The upper part of the figure demonstrates a simplified workflow to extract VPC (ventricular premature contraction) waves from the raw data. The 12 cropped VPC waves will be further stacked together to form a 12 × 1024 matrix for model input. The lower part of the figure demonstrates a simplified structure of the model with feature maps passed through the model. The red square is the kernel of each CNN layer. The number of feature maps and the ratio of feature maps are modified for better demonstrations. C1−C6 is the label for each CNN layer, which matches the maker used in Table 2.

The raw ECG data containing potential recording from 12 leads range from 4 s to nearly one minute. VPC waves are extracted manually by marking out the beginning and end of a single VPC wave as shown in the left upper part of Figure 3. Only non-continuous waves were used for training. Continuous VT waves were excluded to simplify the analysis. As a result, the number of VPC waves extracted from each patient varied, and ranged from one to 20 to feed the CNN model, the dimension of the input is fixed at 12 × 1024. We further extended the tail of the data along the time axis with the last value of recording up to the length of 1024 since not all VPC waves have the same length. For waves with more data points, we trimmed the tail off to fix the length to 1024.

For simplicity, we only include the VPC waves composed of complete QRS and T waves. We excluded the continuous VT, which is difficult to determine the start and end of each excitation. In total, we identified 2518 waves of VAs from 356 patients at Taipei General Hospital, Taiwan. For online data which is gathered from Chapman University and Ningbo First Hospital of Zhejiang University, we identified 1110 waves of VPC from a total of 287 patients. The overall composition of the data is shown in Table 1.

### 2.4. Input Format and Model Structure

As observed from previous clinical experience, the assessment for the source of VPC required more than one lead information. For example, the earlier the precordial transition indicated an RVOT origin. Based on this intuition, we choose to include all signal information from 12 ECG leads for model input. The 12 leads ECG signals are stacked together to form a 2Dimage-like a matrix for model input as shown in Figure 3. The horizontal axis will be the time step, and the vertical axis will be the lead with the order I, II, III, aVR, aVL, aVF, V1, V2, V3, V4, V5, V6, from top to button.

We construct our CNN model from scratch without any pre-trained models. The model is composed of two groups of convolutional neural network (CNN) layers, which is inspired by a previous study [22]. The first group of CNN layers contained a temporal kernel for feature extraction along the time axis of the data. This part of the model contained three blocks. Each block contained convolution, average pooling, and batch normalization. When the data passes through these layers, the vertical dimension stays the same. The second group of CNN layers contained two blocks. The first block contained a cross-leads kernel to extract spatial information. Each block also contained convolutional, average pooling, and batch normalization. The vertical dimension of the data shrinks to one after passing through the first block. This block is followed by another CNN layer with a temporal kernels, as shown in Figure 2. Activation map and fluoroscopic map for ventricular premature complex originating from the right coronary cusp (RCC), the temporal kernel l has a larger size along the time axis while the cross-lead kernel has a larger axis along the lead axis. After the CNN layer, two fully connected layers were used. The sigmoid activation is used for the output from the last layer of the model. Details of the parameter of each layer are shown in Table 2. Dropout layers were used as follows by every convolution layer with 10% the input value set to zero. Batch normalization is used following every average pooling layers.

### 2.5. Data Allocation

We choose around 8% of all the data for model testing and 10% for validation. To make sure the label accuracy, we only used data from the Taipei Veteran General Hospital. There are 11 classes of location based on the classification system we use, and can be viewed from Table 1 [21]. For testing data, we did not just randomly select cases from the total data set. We specifically picked cases from all of the 11 classes to generate testing data set that has a more even distribution of cases. Otherwise, certain classes might not have a case to be tested. To test data as variable as possible, we randomly selected cases from the class with more than two cases. The distribution of the selected cases is shown in Table 1. We also intentionally increased the number of cases over LV summit, since these testing data are also used for testing the model for distinguishing LV summit. To prevent imbalanced testing data, we specifically chose the data with VPC from different heart ventricles. The result is that the VPC from the left and right side of the heart is 49.1% and 50.9% (by patient number).

### 2.6. Model Training

#### 2.6.1. For Binary Classifying Right- and Left-Sided VPC Source

With the model structure described above, we trained the first model for binary classification of VPC sources from the left or right side of the chamber. The training and testing data sets are shown in Table 1. For class prediction, a model output value closer to zero will be classified as the left side.

#### 2.6.2. For Classifying Summit of Ventricle from Other VPC Sources

We further trained a model using the same structure to identify VPC arising from the LV summit. We used the same training and testing data set as shown in Table 1, but we modified the label to separate the left ventricle summit from the rest of the other locations. For class prediction, a model output value closer to one will be classified as a left ventricle summit.

#### 2.6.3. Hyperparameter Tuning, Training Policy, and Other Training Methods Used

Hyperparameters such as batch size, number of convolution layers, and number of neurons in the fully connected layers were tuned based in the performance of the model on the validation data set. For the training rate schedule, a cyclic training rate with exponential decay is used [23]. The step size was set at 32 with base learning of 1 × 10^−4^ and maximum learning of 0.01. To prevent overfitting, early stopping policy is applied. We tolerated up to four epochs if the loss of the validation data was higher than the training data.

Most idiopathic VPCs usually arise from the RVOT (70–80%). LVOT only accounts for less than 20%. For the imbalanced data issue, we applied weighted sampling to increase the chance of VPC arising from the 11 different sub-classes. For classifying the summit of the ventricle from another VPC source, we also performed weighted sampling to amplify the ratio of VPC arising from the summit being used for training.

For overfitting, we applied data augmentation by randomly stretching the wave from 0 up to 20%. Signals from each lead were also vertical drifted randomly from −200 mV to 200 mV. VPC waves were randomly trimmed from the tail and head up to 100 steps.

### 2.7. Model Evaluation

The receiver operating characteristic curve (ROC) is used for model evaluation. The accuracy, F-score, sensitivity, specificity, and positive-predict value are also provided. To further identify the source of error, we performed subgroup analysis by plotting the distribution of model output from different VPC sources. The distribution is shown with a violin plot.

### 2.8. Combining Two Models for VPC Site Identification

We further combined the model for left-right classification and LV summit classification to test the potential in a clinical scenario. Thresholds were chosen to obtain the best g-mean for both models. Testing data were first classified by model for left-right ventricle source identification. For the cases classified on the left side, they will be further processed with a second model to establish whether it is from the LV summit.

### 2.9. Implementation of Model Building, Training, and Model Evaluation

The model was trained with NVIDIA RTX 3070 GPU. The framework used for model building, training, and testing was made with Pytorch 2.0 [24]. The training time for each epoch was around 5 s. Further evaluation with Receiver operating characteristic (ROC) curve and violin plots were constructed with scikit-learn library in conjunction with matplotlib.

## 3. Results

### 3.1. Study Population

Our study merged data from two datasets. The first one is an open-source data set from Chapman University and Ningbo First Hospital of Zhejiang University, China, which consisted of VPC ECG recordings from 334 patients. The second is from Taipei Veterans General Hospital, Taiwan. Table 3 shows the baseline characteristics of the enrolled study population from Taipei Veterans General Hospital. The mean age was 49.6 ± 15.6 and 49.8% of patients were male in patients from Taipei Veterans General Hospital. Hypertension was the most common underlying disease, followed by dyslipidemia and type 2 DM. More details of the other open-source data sets can be found in the report of J Zheng et al. [17].

### 3.2. Training and Testing Data Descriptions

Details of the VPC source distribution over the raining and testing datasets are shown in Table 1. Left ventricular outflow tract (LVOT) and RVOT combined account for the largest portion of the training data, which is about 70%. For locations outside the outflow tract, they account for the remaining 30%. For the ratio between location over left or right ventricle, 49.1% is from the left side, and 50.9% is from the right side (by patient number).

### 3.3. Model Performance for Classifying Right- and Left-Sided VPC Source

**Figure 4:**
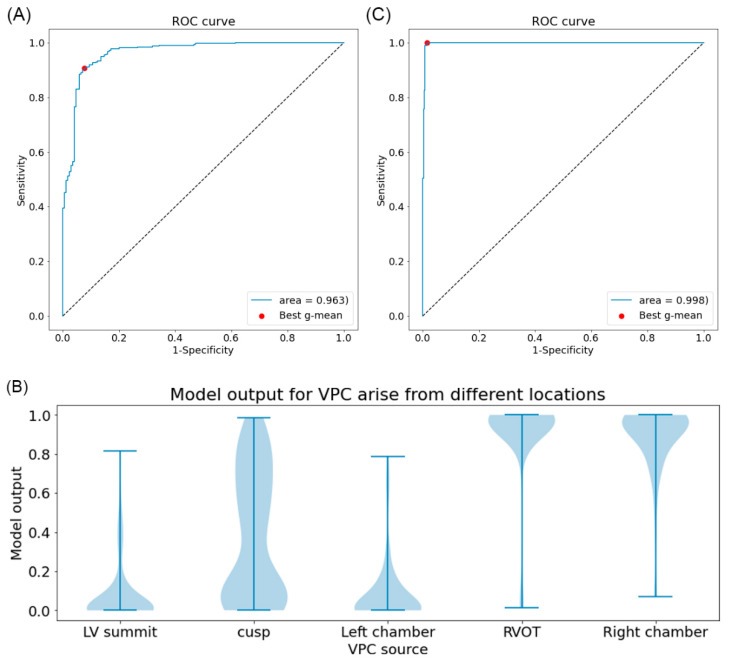
Receiver operating characteristic (ROC) of models for classifying ventricular premature contraction and distribution of model output from a different location. (**A**) The ROC of the model for distinguishing VPC from the left or right ventricle. The red dot indicates the best geometrical mean (g-mean) of sensitivity and specificity. (**B**) The vertical line of each group indicates the range of the values. The width of the plot indicates the ratio of data with this value. (LV summit: VPC from left ventricle summit, Cusp: VPC from right coronary cusp, left coronary cusp, right and left cusp junction. Left chamber: VPC from the aorto-mitral curtain, Left anterior fascicle, mitral annulus, left ventricular papillary muscle, RVOT: VPC from right ventricle outflow tract. Right chamber: VPC from right ventricular parahisian, pulmonary artery, right ventricular moderator band, tricuspid annulus). (**C**) The ROC of the model for identifying VPC from the summit of the left ventricle. The red dot indicates the best geometrical mean (g-mean) of sensitivity and specificity.

After training, we obtain a model with an area under the curve of ROC of 0.963. (Figure 4A). The threshold of the best geometric mean (g-mean) between sensitivity and specificity is 0.739. With this threshold, the sensitivity and specificity are 90.7% and 92.3% for identifying left side VPC. In addition, for accuracy and F1-score, they are 0.91 and 0.88 respectively. Further subgroup analysis, a violin plot is shown in Figure 4B. All the model output from different VPC locations shows a range between 0.0–1.0, which means there were always certain misdiagnosed cases regardless of the threshold chosen. The VPC from coronary cusps shows a suboptimal distribution with most of the cases evenly distributed across values ranging from 0.0 to 1.0. This result indicates that most of the errors in the prediction come from the VPC of the cusp region.

### 3.4. Model Performance for Identifying VPC from LV Summit

The performance of the model for predicting VPC from the LV summit shows excellent results (Figure 4C). The area under the ROC curve is 0.998. The threshold of the best geometric mean (g-mean) between sensitivity and specificity is 0.699, which has a sensitivity of 100% and specificity of 98% for LV summit detection. In addition, accuracy and F1-score were 0.99 and 0.99 respectively.

### 3.5. Data Size and Model Performance

**Figure 5:**
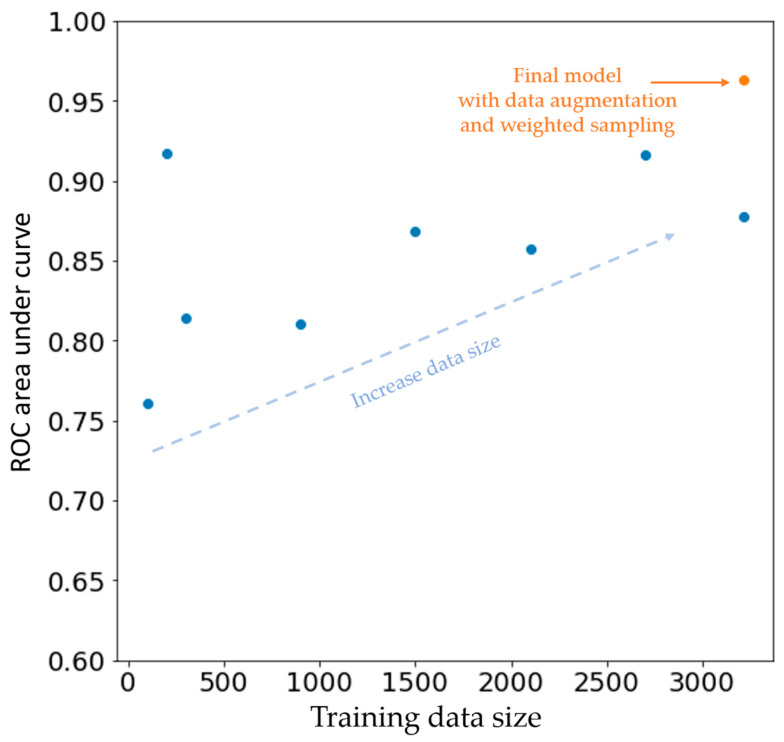
Change in model performance with the increasing size of training data. Each blue dot indicates model performance with a different training data size. The orange dot indicates the final model with the implementation of weighted sampling and data augmentation. The vertical axis is the area under the curve of the ROC from a different model and the horizontal axis is the data size being used. The unit of the data set shown here is one single VPC wave.

To evaluate the effect of data size on the model performance, we train models for Left-Right classification with different data sizes. We observed a trend of increasing model performance with the increase in data size. An early stopping policy was applied and validation data size was fixed. In addition, with the implementation of weighted sampling and data augmentation, the accuracy much improved, as shown in Figure 5.

### 3.6. Combining Model for Left-Right Classification and LV Summit Identification

**Figure 6:**
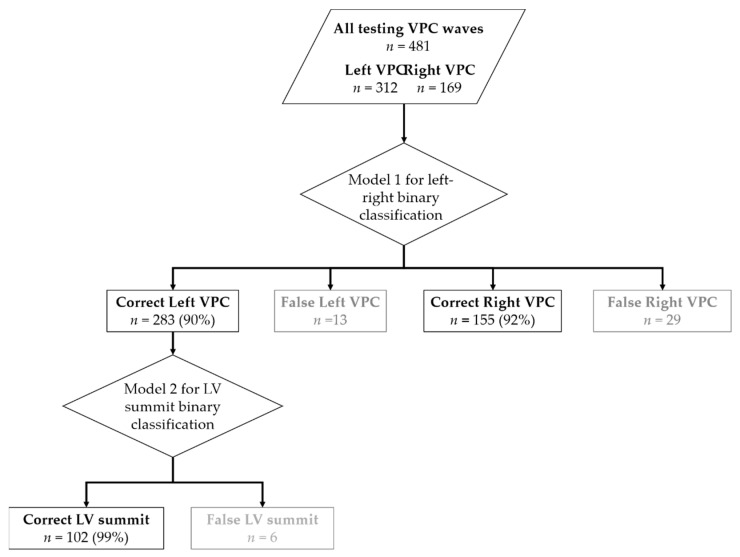
Combining two models for Ventricular Premature Contraction source identification. This workflow shows the performance of combining the use of two models. The percentage shown in the figure indicates the portion of waves being correctly identified. Text and frame with light color indicate false prediction.

We further combined the first and second models into a diagnostic workflow as shown in Figure 6. The positive prediction rate for left side VPC and right side VPC were 90% and 92%, respectively. As for the positive predicted rate of LV summit, it is 99%.

## 4. Discussion

### 4.1. Previous Studies of Manual Measurement and Machine Learning in the Localization of Ventricular Arrhythmia

Several ECG parameters were proposed to distinguish the origin of idiopathic ventricular arrhythmia (Table 4). In 2007, Yang et al. found that the earliest onset or first peak/nadir in V2 and early initial peak/nadir in the inferior leads suggested an RVOT focus [25]. Yamada et al. demonstrated that a right bundle-branch block, transition zone, R-wave amplitude ratio in leads III to II, Q-wave amplitude ratio in leads aVL to aVR, and S waves in lead V6 could predict the origin of LV summit [26]. Later, the transitional zone index was introduced to be a more useful marker for differentiating RVOT origin from left coronary cusp origin [27]. One previous study showed that the V2S/V3R index outperformed other ECG criteria to differentiate left from right outflow tract origins independent of the site of the precordial transition [28]. Furthermore, He et al. established an ECG diagnostic model that consisted of two ECG algorithms-the transition zone (TZ) index and V2S/V3R index, with a sensitivity of 90% and a specificity of 87% [8]. Although these studies have favorable accuracy, the sample size and reproducibility of manual ECG measurement could impact the results.

As artificial intelligence (AI) emerged from the surface, machine learning and deep learning have been applied in the identification of ECG features, as shown in Table 4. Recent studies showed that support vector machines (SVM) could be used in clinical settings to automatically analyze ECG data before and during the procedure [29,30]. By comparison, Zheng et al. demonstrated that CNN supplied with ECG features extraction could attain clinical-grade precision of prediction for localizing the origin of ventricular arrhythmia [31].

### 4.2. The Current Study in Comparison with Previous Artificial Intelligence Studies

Since the data for idiopathic ventricular arrhythmia is very limited, the data size for the previous study is usually small, ranging from a few dozen up to one hundred [4]. Studies that have a few hundred cases are very limited. This is probably why we could only find only a few studies trying to solve the VPC localization problem with machine learning and deep learning at present.

One study group from China (the one from which we obtained the open data), combined a designed featured extraction method and use a Support Vector Machine (SVM) to classify left and right outflow tract ventricular tachycardia [31]. The features being measured included the height of each wave from different leads, the width of each wave, and more. With a training data size (patients) of 340 and a testing data size of 42. The model showed high accuracy and a high F1-score (97.62 and 98.46). However, the real performance of the method in the real world is still questionable since their testing dataset is relatively small and seriously biased to RVOT with only nine LVOT cases, amounting to 42 patients. Another study used a very similar study design to ours that use the CNN model to distinguish left from right ventricle VPC [29]. However, they used 1D-CNN rather than a 2D-CNN. They attach a single VPC wave signal from 12 leads head to tail into a 1-dimensional signal. Then, they trained a one-dimensional CNN model and SVM with a data size of 77 patients and test on 21 subjects. With this setting, they achieved accuracy and F1-score of 0.94 and 0.94 for SVM. For CNN, the accuracy and F1-score are 0.87 and 0.87. They also have a relatively small data set of 21 patients. VPC from the left side only accounts for 29%.

In comparison with these two studies, one of our major advantages is that we have the largest and most balanced testing data set, which can provide a more accurate assessment of the model. We also developed a model that can identify VPC from the summit area with very high accuracy, which can be of high clinical application, comparing only separating left from right VPC. This is probably a benefit of our larger dataset.

Another advantage of our study is that our training data are mainly clinical VA and we only selected solitary VPCs other than VT. Compared to previous studies, some of the training data are based on intraprocedural pacing waveforms [32]. The pace mapping was shown to be misleading, especially in the outflow tract VPC because of the capture of adjacent myocardium or the presence of preferential conductions. Therefore, the application of pacing waveforms for training might fail to predict the VPC origin accurately [33,34,35].

In line with previous studies [31,32], our model showed promising results in differentiating locations of VPC, especially the LV summit. In the subgroup analysis with the violin plot, most VPCs could be classified into the right or left side accurately. However, the result for VPC from coronary cusps was barely satisfactory. The reason for a possible reduction the accuracy of ECG predictive algorithms in the coronary cusp is that VPCs from this region have preferential conduction to the RVOT, possibly because of myocardial fiber orientation. Parts of the coronary cusps are adjacent to the RVOT [36]. Yamada et al. [37] demonstrated that nearly 25% of patients with VAs originating from the coronary cusps, where the earliest activation was located, had better pace mapping results in RVOT in comparison to coronary cusps. When mapping the RVOT, the finding of early but far-field signals may be the consequence of preferential conduction from a deeper LVOT focus.

Furthermore, it was reported that 4% of patients with outflow tract VAs might have a change in preferential conduction that was observed with alteration of the VPC morphology following catheter ablation [38]. The phenomenon was suggestive of an intramural origin of VPC with a shift in the surface breakout due to ablation at the first preferential exit. In this situation, ablation at RVOT and coronary cusp would be needed occasionally.

During the development of the model, we tried to build a multiclass model to classify VPC sources directly into five groups (LV summit, coronary cusp, LV chamber, RVOT, and RV chamber). However, with the same CNN model structure by only replacing the last output layer, we failed to train a model that has comparable performance to the binary classification model. This might be due to a lack of data, or a more complex model is required. This will require further study.

### 4.3. Application of Current Findings into Clinical Practice

The accurate prediction of VA origin before the procedure is crucial in the clinical setting. In terms of VA from the right and left chambers, accurate prediction could contribute to avoiding unnecessary catheter manipulation and therefore reduces the procedure time and perioperative adverse events. In terms of VA from the LV summit, catheter ablation is challenging. The origin of these arrhythmias may be the epicardium of the left outflow tract or the intramural myocardium next to the basal septum, and mapping of the proximal septal venous perforator can help to make this differentiation. VPCs from the LV summit can be eliminated by ablation from the coronary venous system or from adjacent endocardial structures, including septal right ventricular outflow tract, the left coronary cusp, or the basal endocardium of the left ventricle. In some challenging cases, bipolar radiofrequency ablation or ethanol infusion into the coronary venous system was needed due to deeper intramural lesions [39,40]. Thus, accurate prediction of VPC from LV summit before the procedure would be helpful for the operator in preparation, mapping, and ablation.

In conclusion, although the source of VPCs prior to ablation can be predicted from an electrocardiogram, it is sometimes misleading. Our study showed the promising perspective of the machine learning models that predict the origin of VPC. Further model training with accumulated samples is warranted to improve the diagnostic efficacy.

## 5. Study Limitations

First, because we did not have enough well-labeled data to feed a deep learning model, the algorithm currently only predicts the right, left chamber, and LV summit. Second, some conditions, such as cardiomyopathies, reentrant VT, coronary heart disease, and prior structural and congenital abnormalities, are excluded from the study. Thus, the algorithm potentially has a limitation if applied in such scenarios. Third, the data used for training and testing is still relatively small. The dataset might not cover people with different anatomical differences, such as different heart locations, different torso shapes, body sizes, different body muscles and fat composition, which are all known to affect heart potential measurements from the body surface. Fourth, we only enrolled the Asian-pacific population, so our algorithm might not be able to apply to different populations. In the future, our algorithm needs to be evaluated for online detection of VPCs and other ventricular arrhythmias.

## 6. Conclusions

To the best of our knowledge, currently, this is the largest cohort for machine learning to localize VAs. Based on surface ECG signals, a non-invasive method is helpful to facilitate VA localization. The proposed method could be important to optimize ablation strategy and may help to improve ablation outcomes.

## Figures and Tables

**Table 1:** Composition of Ventricular Premature Contraction source of training/validation data and testing Data.

Locations	Training/Validation Data		Testing Data		-	Training/Validation Data		Testing Data	
	Subject Number (TPE/ZJ)%	Wave Number (TPE/ZJ)%	Subject Number TPE (%)	Wave Number TPE (%)	Detailed Location	Subject Number (TPE/ZJ) %	Wave Number (TPE/ZJ) %	Subject Number TPE (%)	Wave Number TPE (%)
LVOT Cusp	111 (40/71) 18.88	750 (448/302) 24.23	15 (27.27)	139 (28.90)	Supravalvular LCC	56 (17/39) 9.52	389 (232/148) 10.70	6 (10.90)	49 (10.19)
					Supravalvular RCC	15 (8/7) 2.55	108 (76/32) 2.98	3 (5.45)	24 (4.99)
					Supravalvular LCC/RCC junction	20 (13/7) 3.40	172 (138/34) 4.74	5 (9.09)	62 (12.89)
					Infravalvular AMC	18 (0/18) 3.06	88 (0/88) 2.43	1 (1.82)	4 (0.83)
					Supravalvular Septo-parahisian	2 (2/0) 0.0	2 (2/0) 0.06	0 (0.0)	0 (0.0)
LV Summit	69 (64/5) 13.73	641 (625/16) 17.67	10 (18.18)	103 (21.41)	LVOT Epicardial AIV/CGV	69 (64/5) 11.73	641 (625/16) 17.67	10 (18.18)	103 (21.41)
LV chamber	24 (22/0) 3.74	199 (199/0) 5.49	3 (5.45)	70 (14.55)	MA	4 (4/0) 0.68	12 (12/0) 0.33	1 (1.82)	55 (11.43)
					PPM Anterolateral	6 (6/0) 0.68	28 (28/0) 0.77	0 (0.0)	0
					PPM Posteromedial	1 (1/0) 0.17	10 (10/0) 0.28	1 (1.82)	9 (1.87)
					Crux	1 (1/0) 0.17	4 (4/0) 0.11	0 (0.0)	0
					Fascicular Left posterior fascicle	3 (3/0) 0.51	14 (14/0) 0.39	0 (0.0)	0
					Fascicular Left anterior fascicle	9 (9/0) 1.53	131 (131/0) 3.60	1 (1.82)	6 (1.25)
RVOT	226 (135/91) 38.44	1273 (930/343) 35.09	17 (30.91)	99 (20.58)	RVOT	226 (135/91) 38.44	1273 (930/343) 35.09	17 (30.91)	99 (20.58)
RV chamber	167 (40/120) 27.21	765 (316/449) 21.09	10 (18.18)	70 (14.55)	Parahisian	2 (2/0) 0.34	2 (2/0) 0.06	1 (1.82)	1 (0.21)
					TA	13 (13/0) 2.21	99 (99/0) 2.73	3 (5.45)	4 (0.83)
					PA	144 (24/120) 24.49	645 (196/449) 17.78	4 (7.27)	24 (4.99)
					PPM	1 (1/0) 0.17	19 (19/0) 0.52	2 (3.63)	41 (8.52)
total	588	3628	55	481	-	588 (301/287)	3628 (2518/1110)	55	481

The composition of data use for training and validation and testing data are shown. The left half of the table shows the cluster’s location of VPC with five different groups. The right side of the table shows a more detailed VPC location. TPE: Data from Taipei General Hospital, ZJ: Data from Chapman University and Ningbo First Hospital of Zhejiang University. The shaded column indicated training data.

**Table 2:** Detailed parameter of the model used in this study.

Marker	Input Size	Layer	Output Size	Number of Feature Maps	Kernel Size	Stride	Activation
-	-	ECG in 2D	12 × 1024	-	-	-	-
C1	12 × 1024	Convolution	16 × 12 × 1024	16	1 × 129	1	ReLU
C2	16 × 12 × 1024	Convolution	16 × 12 × 1024	16	1 × 129	1	ReLU
-	16 × 12 × 1024	Average pooling	16 × 12 × 512	16	-	2	-
C3	16 × 12 × 512	Convolution	16 × 12 × 512	16	1 × 65	1	ReLU
-	32 × 12 × 512	Average pooling	16 × 12 × 256	16	-	2	-
C4	32 × 12 × 256	Convolution	32 × 12 × 256	32	1 × 33	1	ReLU
-	64 × 12 × 128	Average pooling	64 × 12 × 64	64	-	2	-
C5	128 × 12 × 64	Convolution	128 × 1 × 64	128	12 × 1	1	ReLU
C6	128 × 1 × 64	Convolution	128 × 1 × 64	128	1 × 3	1	ReLU
-	128 × 1 × 64	Average pooling	128 × 1 × 64	128	-	2	-
-	1 × 8192	Fully connected	1 × 1024	-	-	-	ReLU
-	1 × 1024	Fully connected	1	-	-	-	Sigmoid

**Table 3:** Clinical characteristics of the study population.

Clinical Features	Taipei Veterans General Hospital (*n* = 397)	Chapman University and Ningbo First Hospital of Zhejiang University (*n* = 334)
Age (years)	48.7 ± 15.6	46.1 ± 13.1
Male (*n*,%)	173 (43.6%)	104 (32%)
Dyslipidemia (*n*,%)	43 (10.8%)	-
Diabetes mellitus (*n*,%)	29 (7.3%)	-
Hypertension (*n*,%)	85 (21.4%)	-
Chronic Kidney Disease (*n*,%)	5 (1.3%)	-
Old stroke (*n*,%)	3 (0.8%)	-
Atrial Fibrillation (*n*,%)	13 (3.3%)	-
OSAS (*n*,%)	8 (2.1%)	-

**Table 4:** Comparison with previous studies for localization of Idiopathic Ventricular Arrhythmias.

Method Type	Methods	Classification	Cases for Testing	Accuracy Sensitivity/Specificity %	Reference
**Deep Learning**	CNN	Left vs. Right side	55	91/92	Current Study
**Deep Learning**	CNN	LV summit vs. others	55	100/98	Current Study
**Deep Learning**	CNN	Left vs. Right side	21	100/92	Ref. [29]
**Machine Learning**	SVM	Left vs. Right side	21	100/82	Ref. [29]
**Machine Learning**	SVM	LVOT vs. others	117	64/? *	Ref. [30]
**Machine Learning**	ECG Feature extraction + SVM	LVOT vs. RVOT	42	96/100	Ref. [31]
**Manual Rules**	RBBB pattern, aVL/aVR amplitude ratio and S wave in V5 or V6	LV summit vs. others	27	87/100	Ref. [26]
**Manual Rules**	The earliest onset of QRS and peak/nadir in V2	LVOT vs. RVOT	45	92/88	Ref. [25]
**Manual Rules**	Combined TZ index and V2S/V3R	LVOT vs. RVOT	695	90/87	Ref. [8]
**Manual Rules**	V2S/V3R index ≤1.5 predicting LVOT origin	LVOT vs. RVOT	207	89/94	Ref. [28]
**Manual Rules**	Transition zone index <0 predicting LVOT origin	LVOT vs. RVOT	112	88/82	Ref. [27]

Left vs. Right side: Indicate that the VPC being studied in this study includes the location of all the ventricles not only the outflow tract. LVOT vs. RVOT: Indicate that the study only focuses on the classification of outflow tract VPC. * Due to the study design, the specificity is unknown. Ref.: Reference.

## Data Availability

The original contributions presented in the study are included in the article/supplementary materials, further inquiries can be directed to the corresponding author.
